# Pharmacologic Targeting of BET Proteins Attenuates Hyperuricemic Nephropathy in Rats

**DOI:** 10.3389/fphar.2021.636154

**Published:** 2021-02-16

**Authors:** Chongxiang Xiong, Jin Deng, Xin Wang, Xiaofei Shao, Qin Zhou, Hequn Zou, Shougang Zhuang

**Affiliations:** ^1^ Department of Nephrology, The Third Affiliated Hospital of Southern Medical University, Guangzhou, China; ^2^ Department of Nephrology, Shanghai East Hospital, Tongji University School of Medicine, Shanghai, China; ^3^ Department of Medicine, Alpert Medical School and Rhode Island Hospital, Brown University, Providence, RI, United States

**Keywords:** bromodomain and extra-terminal proteins, hyperuricemic nephropathy, I-BET151, renal fibrosis, epithelial-to-mesenchymal transition, inflammation

## Abstract

Hyperuricemia is an independent risk factor for renal damage and promotes the progression of chronic kidney disease. In this study, we investigated the effect of I-BET151, a small-molecule inhibitor targeting the bromodomain and extraterminal (BET) proteins, on the development of hyperuricemic nephropathy (HN), and the mechanisms involved. Expression levels of bromodomain-containing protein 2 and 4, but not 3 were increased in the kidney of rats with HN; administration of I-BET151 effectively prevented renal dysfunction, decreased urine microalbumin, and attenuated renal fibrosis as indicated by reduced activation of renal interstitial fibroblasts and expression of fibronectin and collagen I in HN rats. Mechanistic studies show that I-BET151 treatment inhibited transition of renal epithelial cells to a mesenchymal cell type as evidenced by preservation of E-cadherin and reduction of vimentin expression. This was coincident with reduced expression of TGF-β1 and dephosphorylation of Smad3 and ERK1/2. I-BET151 was also effective in inhibiting phosphorylation of NF-κB, expression of multiple cytokines and chemokines, and infiltration of macrophages to the injured kidney. Although there were increased serum levels of uric acid and xanthine oxidase, an enzyme that catalyzes production of uric acid, and decreased expression of renal organic anion transporter 1 and 3 that promote urate excretion in the model of HN, and reduced expression levels of urine uric acid, I-BET151 treatment did not affect these responses. Collectively, our results indicate that I-BET151 alleviates HN by inhibiting epithelial to mesenchymal transition and inflammation in association with blockade of TGF-β, ERK1/2 and NF-κB signaling.

## Introduction

Chronic kidney disease (CKD) is a global public health problem that affects about 10% of the population worldwide (Zhang et al., 2012). Hyperuricemia is an independent risk factor for renal damage and is tightly associated with the development and progression of CKD as well as many other diseases, such as diabetes and hypertension (Johnson et al., 2018; Srivastava et al., 2018). Uric acid is an end metabolic product of purine metabolism and is excreted from the kidney. Hyperuricemia can be caused by increased production of uric acid and decreased urinary excretion of uric acid. Uric acid is derived from dietary and endogenously synthesized purines; xanthine oxidase (XOD) plays a key role in the conversion of hypoxanthine to xanthine and xanthine to urate. The proximal tubule is the site of uric acid reabsorption and secretion. In normal kidney, approximately 90% uric acid is reabsorbed into blood; a portion of uric acid can be secreted into urine though the urate transporters. Organic anion transporter 1 (OAT1) and organic anion transporter 3 (OAT3) have been reported to be the two major transporters involved in uric acid secretion (Otani et al., 2017). Decreased expression of these transporters can lead to accumulation of uric acid in the body, resulting in hyperuricemic nephropathy (HN).

Increasing evidence indicates that hyperuricemia can induce kidney stones and is associated with glomerular hypertension, arteriolosclerosis, and tubulointerstitial fibrosis (Vargas-Santos and Neogi, 2017; Johnson et al., 2018). Renal tubulointerstitial fibrosis is characterized by excessive deposition of the extracellular matrix (ECM) in renal tissue (Wang, et al., 2017) and activation of renal interstitial fibroblasts (myofibroblasts). Myofibroblasts express α-smooth muscle actin (α-SMA) and is the main cell type that produces ECM proteins such as collagen I and fibronectin. Following injury, renal tubular cells with maladaptive repair can convert to a mesenchymal cell type that produces numerous profibrotic cytokines/chemokines, including transforming growth factor β1 (TGF-β1) and monocyte chemoattractant protein-1 (MCP-1) (Lovisa et al., 2015; Stone et al., 2016), interleukin-1β (IL-1β), and tumor necrosis factor-α (TNF-α) (Mulay et al., 2014; Liu et al., 2015). MCP-1 can induce infiltration of macrophages into injured kidneys; TGF-β1 can drive activation of renal fibroblasts and induce renal epithelial-mesenchymal transition (EMT) by activation of Smad3 signaling (Ryu et al., 2013; Meng et al., 2015). Other signaling pathways such as NF-kB and ERK1/2 can also be activated by high uric acid and involved in renal epithelial-mesenchymal transition and the pathogenesis of HN (Liu et al., 2017a; Liu et al., 2017b; Tao et al., 2019).

Studies have shown that the transcription of genes involved in inflammation and other pathological processes in renal diseases are regulated by epigenetic changes (Nangaku et al., 2017; Morgado-Pascual and Marchant, 2018). Epigenetic modifications, in particular histone acetylation, are dynamic processes in normal and diseased states (Reddy and Natarajan, 2015; Morgado-Pascual et al., 2019). It is well documented that histone acetylation is positively regulated by histone transferases and negatively regulated by histone deacetylases (Guo et al., 2019; Tang and Zhuang, 2019). The bromodomain and extraterminal (BET) proteins are epigenetic “readers,” which recognize and bind to acetylated lysine in histones and other proteins and then regulate gene transcription (Morgado-Pascual et al., 2019). Among BET family of proteins, including bromodomain-containing protein (BRD)2, BRD3, Brd4, and the testis-specific BRDT, Brd4 is best studied and has been identified to be in the chronically injured kidney (Xiong et al., 2016; Suarez-Alvarez et al., 2017; Morgado-Pascual et al., 2019). It has been reported that small-molecule inhibitors targeting BET such as I-BET and JQ1can inhibit many pathological processes by competitively binding to acetyl-lysine recognition motifs and disassociating BET proteins from chromatin. Studies have shown that BET inhibitor could ameliorate tissue fibrosis in the lung (Tang et al., 2013; Wang et al., 2018), liver (Ding et al., 2015), heart (Duan and McMahon, 2017) and other sites of inflammation (Hytti et al., 2016; Poghosyan et al., 2016; Suarez-Alvarez et al., 2017). Recently, we and others have demonstrated that abrogating BET with I-BET or JQ1 attenuates renal fibrosis following ischemia/reperfusion, angiotensin II injection or unilateral ureteral obstruction (UUO) (Wilflingseder and Willi, 2020) (Xiong et al., 2016; Wang et al., 2019). However, there are no reports assessing the pharmacological effect of BET inhibitors on the development of HN.

In this study, we investigated the effect of BET protein inhibition with a small-molecule inhibitor, I-BET151, on the development of HN. I-BET151 has a longer half-life in animals (Alqahtani et al., 2019) and is under clinical trial for the treatment of advanced or recurrent solid tumors (NCT02630251). Our results demonstrated that administration of I-BET151 effectively improved renal function and ameliorated renal fibrosis in association with inhibition of TGFβ/Smad3, ERK1/2 and NF-kB signaling and suppression of inflammation.

## Materials and Methods

### Antibodies and Reagents

Antibodies to Brd3, Brd4, fibronectin, p-NF-κB (p65), NF-κB (p65), and CD68 were purchased from Abcam (Cambridge, MA, United States). Antibodies to collagen I, α-SMA were purchased from Affinity Biosciences (Cincinnati, OH, United States). Antibodies to smad3 and p-smad3 were purchased from Hangzhou HuaAn Biotechnology Co., Ltd. (Hangzhou, China). Antibodies to p-ERK1/2 and ERK1/2 were purchased from Cell Signaling Technology (Danvers, MA). Antibodies to E-cadherin, vimentin were purchased from Proteintech (Chicago, IL, United States). Antibodies to glyceraldehyde 3-phosphate dehydrogenase (GAPDH) was purchased from CWBIO (Beijing, China). Brd2 antibody was purchased from Boster Biological Technology (Wuhan, China), OAT1 and OAT3 antibodies were purchased from Santa Cruz Biotechnology (Santa Cruz, CA), I-BET151 was purchased from Target Mol (MA, United States). TNF-α, IL-1β, MCP-1, RANTES, and TGF-β1 ELISA kits were purchased from CUSABIO Technology LLC (Wuhan, China). The serum XOD kit was purchased from Jiancheng Technology (Nanjing, China).

### Animals and Experimental Design

Six to eight week old male Sprague–Dawley (SD) rats, of weight 200–220 g, were purchased from experimental animal center of Southern Medical University, China. The rats were housed in a specific pathogen-free (SPF) laboratory at the Animal Experimental Center of Southern Medical University. The rats were allowed one week to adapt to the environment before experiments. Twenty-four male rats were randomly divided into four groups of six rats: sham group, sham treated with I-BET151 group, HN group, and HN treated with I-BET151 group. The HN rat model was established by oral administration of a mixture of adenine (0.1 g/kg) and potassium oxonate (1.5 g/kg) daily for 3 weeks as described in our previous study (Liu et al., 2015). To examine the efficacy of I-BET151 in HN rats, I-BET151 (5 mg/kg) in 50 μl dimethyl sulfoxide (DMSO) was given via peritoneal injection every day to the rats 1 h after the mixture of adenine and potassium oxonate exposure was taken by rats. For the sham alone group and HN alone groups, rats were injected with an equivalent amount of DMSO. Experimental protocols were approved by the Southern Medical University Experimental Animal Ethics Committee (Guangzhou, China).

### Measurement of Renal Function

At day 21 after feeding a mixture of adenine (0.1 g/kg) and potassium oxonate, all rats were sacrificed and blood was taken for the measurement of serum creatinine, BUN, uric acid, and other biochemistry index by automatic biochemistry assay using kits purchased from Shanghai Roche Pharmaceuticals Lt (Shanghai, China). In addition, urine samples were collected for the measurement of microalbumin and uric acid using the kits purchased from Shanghai Roche Pharmaceuticals Lt (Shanghai, China) as well.

### Histology and Immunohistochemistry

Formalin-fixed kidneys were dehydrated and embedded into paraffin blocks. Then, 3-μm thickness of sections were stained with hematoxylin–eosin (HE) and Masson’s trichrome. Tubular injury was scored on a scale from 0 to 3, where 0 = normal, 1 = injury <30%, 2 = injury 30–60%, 3 = injury >60%. For immunohistochemistry, the sections were transferred into a 10-mmol/L citrate buffer solution (pH = 6.0). Then, the sections were treated in a microwave for 20 min for antigen retrieval. After being blocked with 3% hydrogen peroxide for 20 min at room temperature, the sections were incubated with primary antibodies at 4°C overnight. Subsequently, the sections were washed with PBS three times and incubated with the secondary antibody for 30 min at room temperature. Thereafter, the sections were stained with 3,3′-diaminobenzidine (DAB, Golden Bridge Biotechnology Co., Beijing, China) and then counterstained with hematoxylin. Tubular Score and the assessment of Masson trichrome staining were performed according to our previous study (Liu et al., 2015).

### Western Blotting Analysis

Renal tissues were sufficiently homogenized in RIPA lysis Buffer and total proteins were extracted using a commercial extraction kit (Key GEN Biotech, Nanjing, China). Protein concentrations were determined using the Bicinchoninic Acid (BCA) Protein Assay Kit (Beyotime, Shanghai, China). Equal amounts of total protein were separated in SDS-PAGE gels and transferred to polyvinylidene difluoride (PVDF) membranes (Millipore, Bedford, MA, United States). After blocking in 5% BSA, the membranes were incubated with primary antibodies overnight at 4°C. Thereafter, the immunoreactive bands were washed with TBST, incubated with corresponding secondary antibody (1:5,000, CWBIO) for 60 min at room temperature. Then, immunostained proteins were detected by a digital visualizer (Eastman Kodak Company, United States) using chemiluminescence detection reagents. All experiments were repeated three times.

### Assessment of Serum Activity of XOD

Serum activity of XOD was determined according to the instructions provided by the manufacture (A002, Jiancheng, Nanjing, China).

### ELISA Analysis

Serum cytokine levels were examined using ELISA kits according to the protocol provided by the manufacturer (CUSABIO Technology LLC, Wuhan, China).

### RT-PCR

Total RNA was extracted using TRIzol (TaKaRa, Dalian, China). mRNAs were converted to cDNA using the Transcriptor First Strand cDNA synthesis kit (TaKaRa).

Quantitative real-time PCR was performed using SYBR Green PCR master mix (Applied Biosystems) in a BioRadiCycleriQ Detection System. The primer sequences are as follows: OAT1 forward 5′-GAG​CTG​TAC​CCC​ACA​GTG​ATT-3′, reverse 5′-GAA​CTC​TGC​AGT​CAT​ACT​CAC​C-3′; OAT3 forward 5′-AGT​CCT​CGG​AAT​AGC​CAA​CC-3′, reverse 5′-TGT​ACG​AAG​CGG​AGA​CAC​TT-3′; GAPDH forward 5′-CCT​CGT​CTC​ATA​GAC​AAG​ATG​GT-3′, reverse 5′-GGG​TAG​AGT​CAT​ACT​GGA​ACA​TG-3′.

### Statistical Analysis

Data are expressed as mean ± standard error of mean (SEM) and were analyzed using the SPSS 20 software. Comparisons among groups were performed using one-way ANOVA. *p* < 0.05 were considered statistically significant.

## Results

### Expression of BET Proteins in the Kidney of HN Rats and the Effect of I-BET151

**FIGURE 1:**
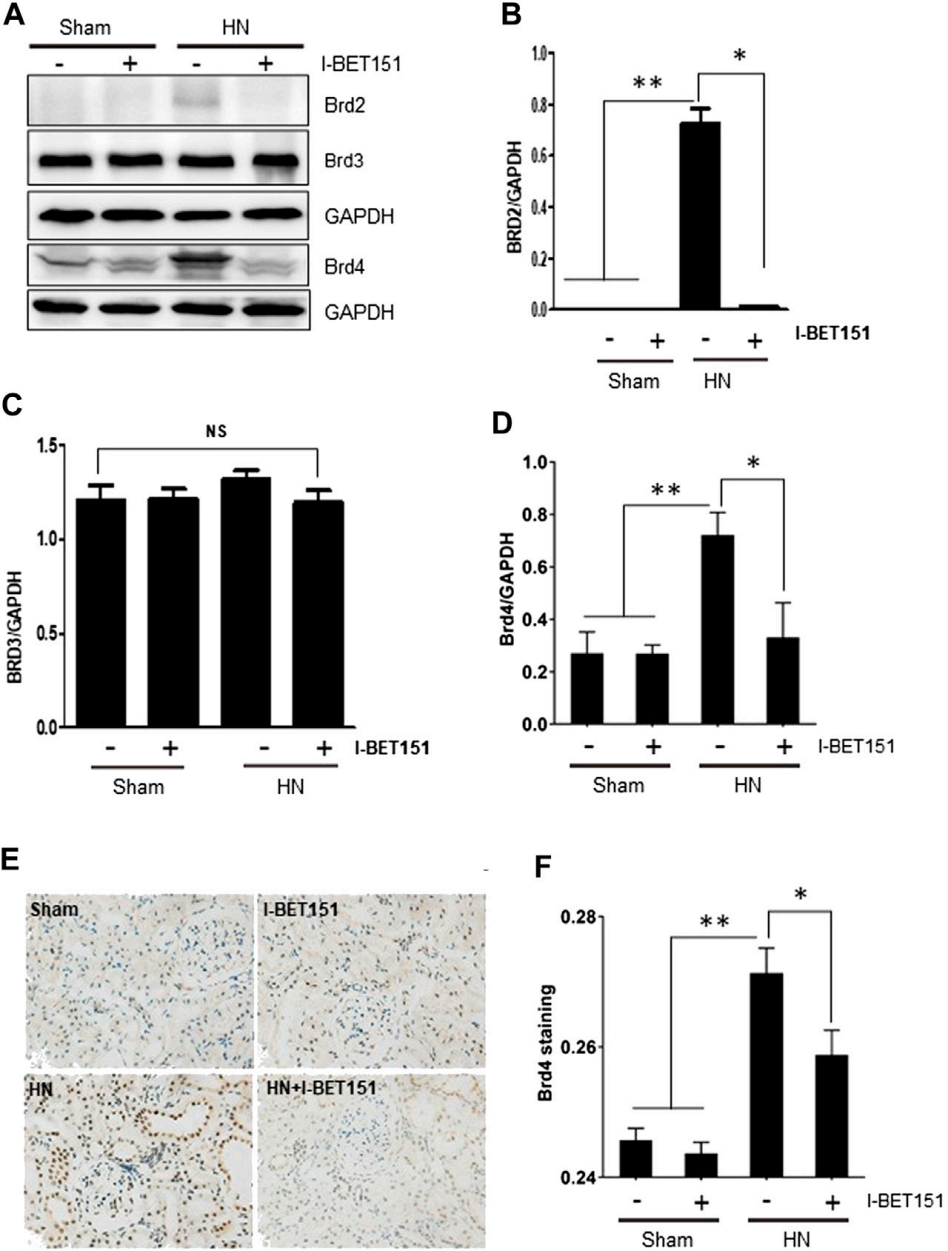
I-BET151 reduces Brd4 levels in the kidney of HN rats. **(A)** A rat model of HN was established and treated with I-BET151 as indicated in “Material and Methods.” After 3 weeks, kidneys were taken for immunoblot analysis for Brd2, Brd3, Brd4 or GAPDH. Expression levels of Brd2 **(B)**, Brd3 **(C)**, Brd4 **(D)** were quantified by densitometry analysis and then normalized with GAPDH. **(E)** Photomicrographs (original magnification, ×400) illustrate immunohistochemical Brd4 staining of kidney tissues. **(F)** Brd4 staining graphic presentation of quantitative data. Data are represented as the mean ± SEM. *p < 0.05; **p < 0.01.

Brd2, Brd3 and Brd4 are three major members mediating a variety of biological responses of the BET family proteins (Morgado-Pascual et al., 2019). As first step toward understanding the effect of I-BET151, a highly selective BET inhibitor, on the pathogenesis of HN, we examined the expression of Brd2, Brd3 and Brd4 in the kidneys of rats with or without HN. As shown in Figures 1A–D, basal levels of Brd3 and Brd4, but not Brd2 were clearly detected in the kidney of rats without HN. Renal expression levels of Brd2 and Brd4 were increased after feeding a mixture of adenine (0.1 g/kg) and potassium oxonate, while administration of I-BET151 abolished their expression. In contrast, Brd3 expression levels remained the same in the kidney of HN and not affected by I-BET151 treatment. Immunohistochemistry staining shows that increased expression of Brd4 was primarily distributed in the nucleus of renal epithelial cells; I-BET151 treatment reduced its expression (Figures 1E,F). Thus, hyperuricemia mainly induces expression of Brd2 and Brd4, which was sensitive to I-BET151 inhibition.

### Administration of I-BET151 Improves Renal Function and Alleviates Proteinuria and Renal Pathology in HN Rats

**FIGURE 2:**
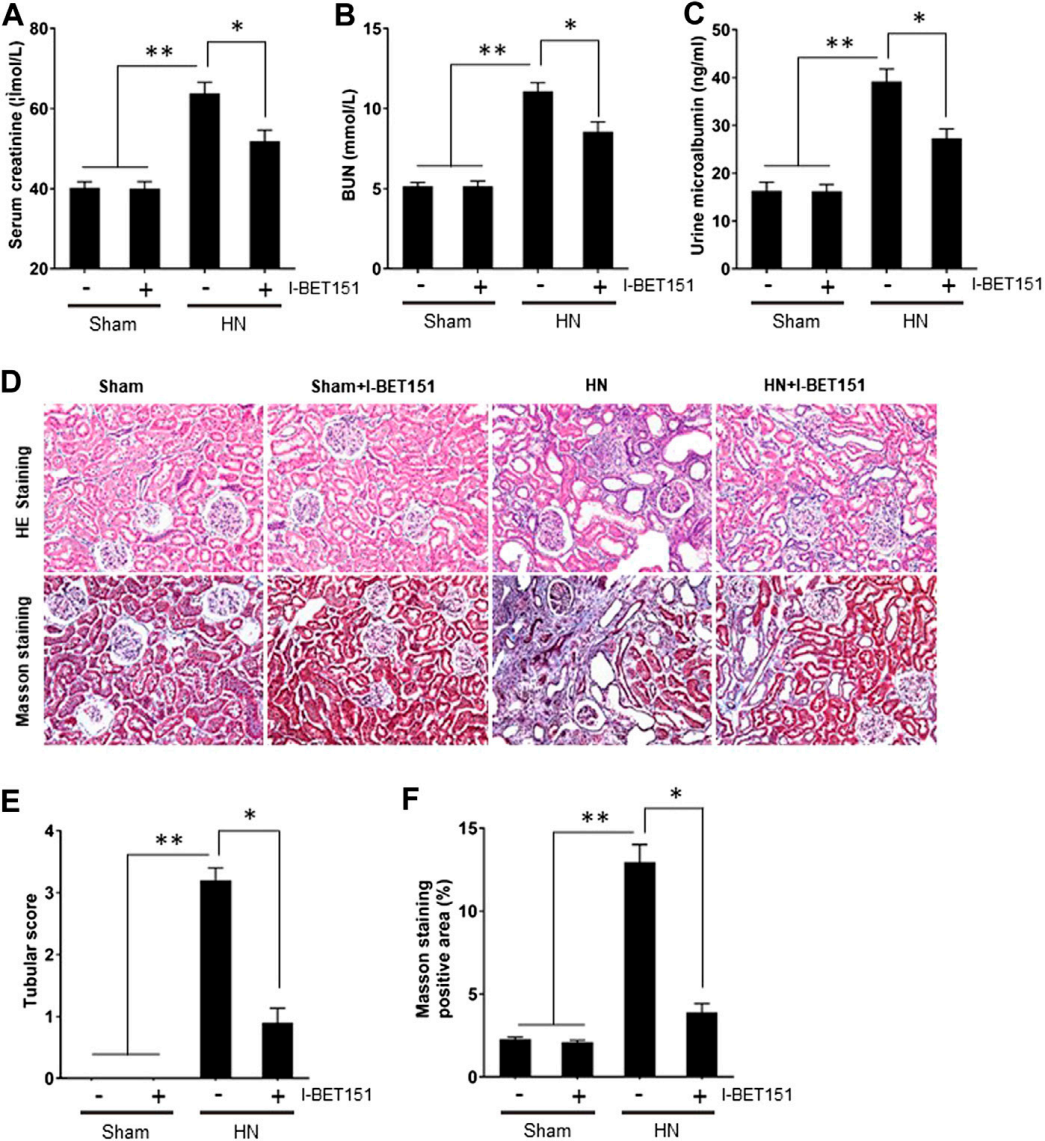
I-BET151 improves renal function and alleviates proteinuria and renal pathology in HN rats. HN rats were treated with I-BET151 for 3 weeks and then blood, urine and kidneys were collected. Levels of **(A)** serum creatinine, **(B)** blood urea nitrogen (BUN), and **(C)** urine microalbumin were examined using biochemical assays. **(D)** Photomicrographs illustrating hematoxylin–eosin (HE) and Masson’s trichrome staining of kidney tissue from the different groups (original magnification ×200). **(E)** Tubular changes were scored as described in “Materials and Methods.” **(F)** The graph shows the percentage of Masson-stained tubulointerstitial area (blue) relative to the whole area from 10 random cortical fields (original magnification×400). Data are represented as the mean ± SEM. *p < 0.05; **p < 0.01.

To demonstrate the functional role of BET proteins in HN, we assessed the effect of I-BET151on renal function and proteinuria as well as renal pathological changes in HN rats. As shown in Figure 2, levels of serum creatinine, blood urea nitrogen (BUN) and urine microalbumin were increased in the HN rats comparted with control rats; I-BET151 treatment significantly reduced the levels of serum creatinine, BUN and urine microalbumin (Figures 2A–C). In addition, HE staining revealed glomerulosclerosis, tubular atrophy, tubular dilatation, and inflammatory cell infiltration in the kidney of rats with HN (Figures 2D,E). I-BET151 treatment also reduced these pathological changes.

Because renal interstitial fibrosis is the common pathological feature of all types of CKD, including HN (Eddy, 2014; Liu et al., 2015; Meng et al., 2016; Liu et al., 2017b), we examined the effect of I-BET151 on the development of renal fibrosis in rats with HN. Masson’s staining demonstrated that HN rats had severe renal interstitial fibrosis as indicated by expanded interstitium and deposition of fibrillar collagens in the kidney (Figures 2D,F). Administration of I-BET151 largely reduced these pathological changes.

Collectively, these results indicated that I-BET151 can effectively improve renal function and attenuate renal histological damage and renal fibrosis in HN rats.

### I-BET151 Inhibits Renal Fibroblast Activation and Attenuates Expression of ECM Proteins in the Kidney of Rats with HN

**FIGURE 3:**
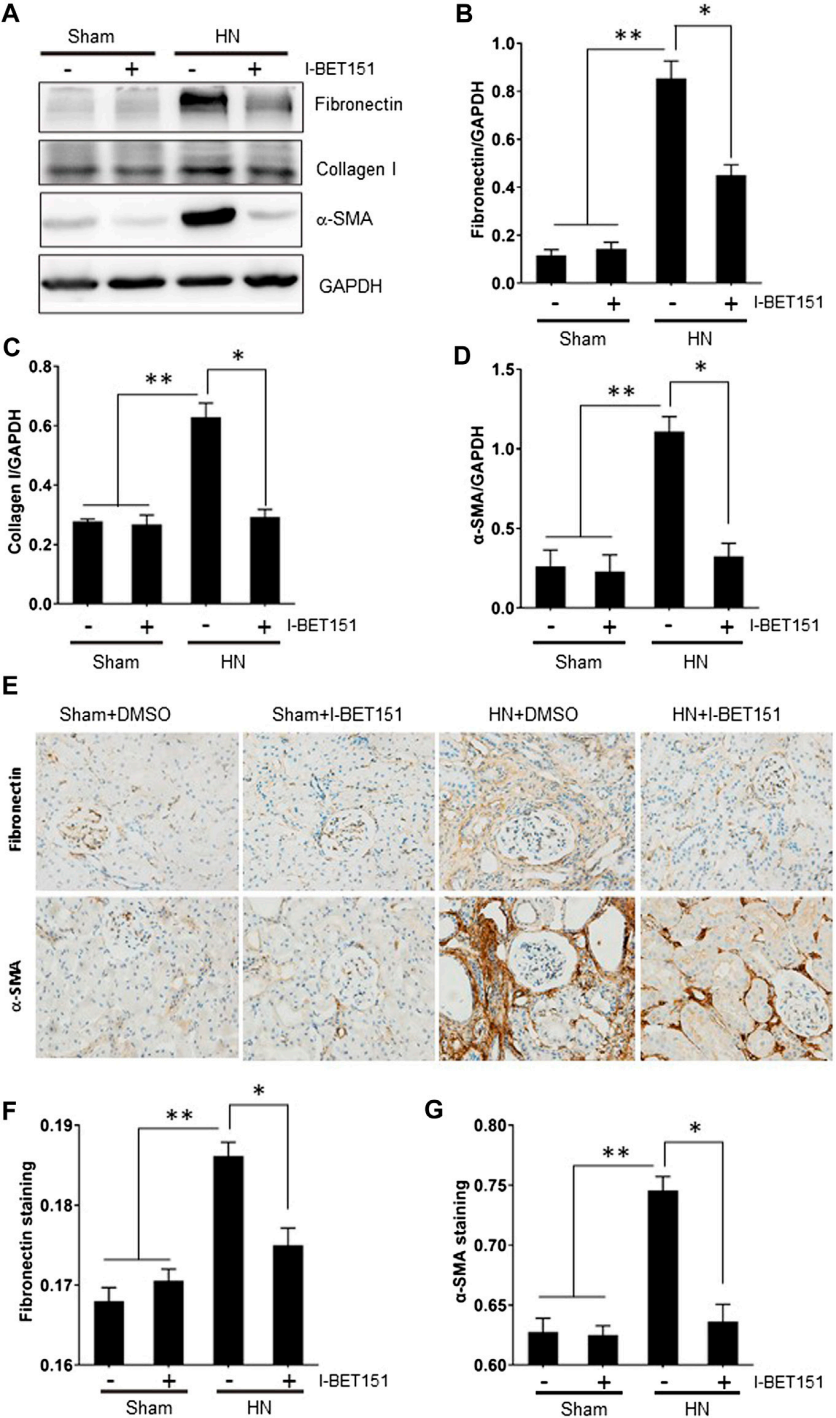
I-BET151 treatment reduces expression of fibronectin, collagen I and α-SMA in kidneys of HN rats. **(A)** Kidney tissue lysates were subjected to immunoblot analysis with specific antibodies against α-SMA, fibronectin, collagen I, or GAPDH. Expression levels of fibronectin **(B)**, collagen I **(C)**, α-SMA **(D)** were quantified by densitometry and normalized with GAPDH. **(D)** Photomicrographs (original magnification ×400) illustrate immunohistochemical fibronectin staining of kidney tissues. **(E)** Photomicrographs illustrating Fibronectin and α-SMA immunochemical staining of kidney tissue. Fibronectin **(F)** and α-SMA **(G)** positive areas relative to the whole area was quantified. Data are represented as the mean ± SEM. *p < 0.05; **p < 0.01.

To confirm the role of BET proteins in mediating renal fibrosis, we further examined the effect of I-BET151 on renal fibroblast activation and expression of ECM proteins by immunoblot analysis and immunochemistry. As shown in Figure 3A, rats with HN displayed an increase in the expression of α-SMA, a hallmark of myofibroblasts (active fibroblasts) in the kidney and I-BET151 abolished this response. Similarly, expression of collagen 1 and fibronectin, two ECM components, was also upregulated in the kidney of hyperuricemic rats, and I-BET151 treatment reduced their expression. Immunochemistry demonstrated similar inhibition of I-BET151 on the expression of fibronectin and α-SMA. Taken together, these data illustrated that I-BET151 is a potent inhibitor that inhibits renal fibroblast activation and accumulation of ECM proteins in kidneys of HN rats.

### I-BET151 Inhibits Renal Tubular Epithelial-to-Mesenchymal Transition in the Kidney of HN Rats

**FIGURE 4:**
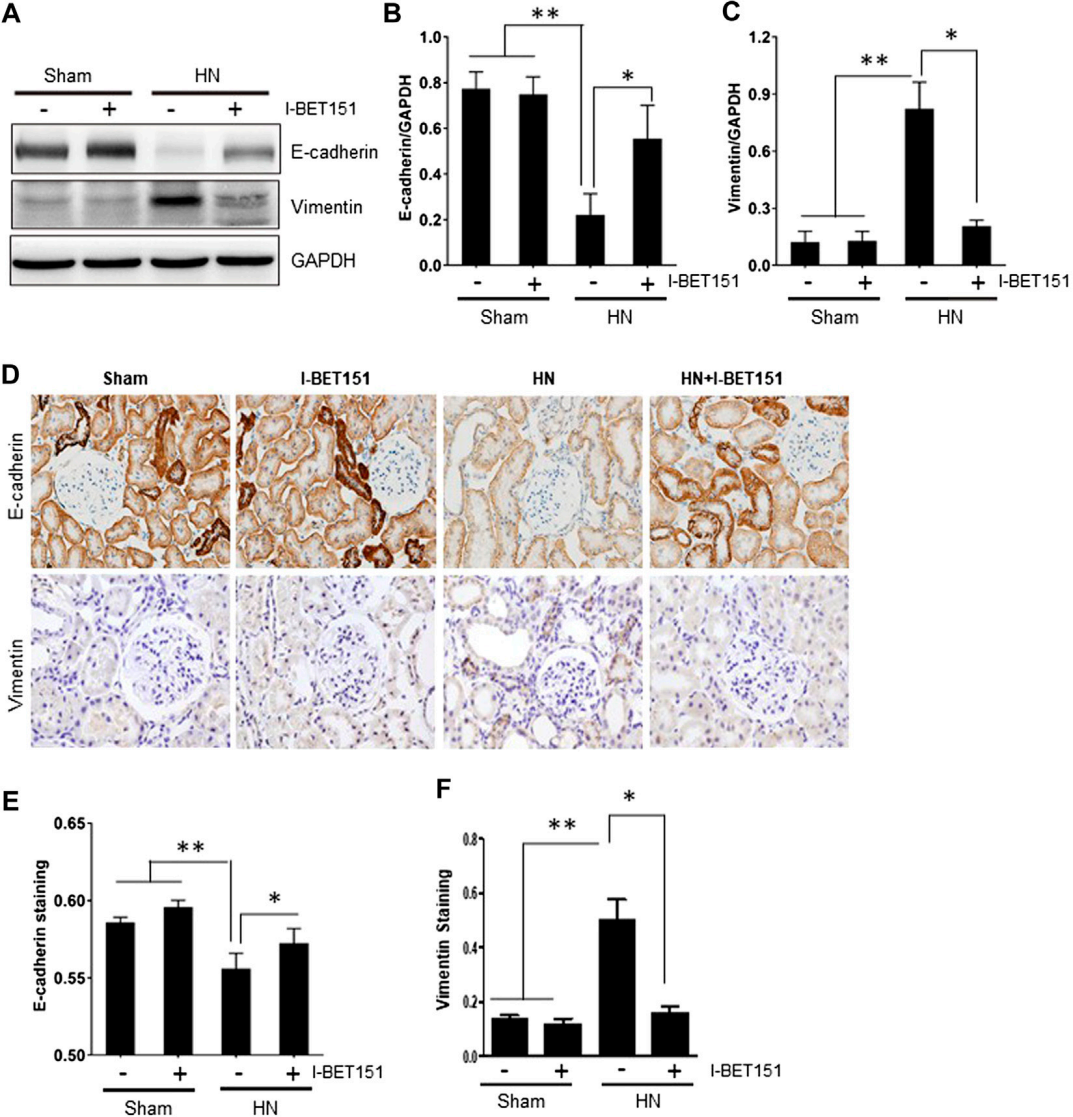
I-BET151 inhibits renal tubular epithelial-to-mesenchymal transition in the kidney of HN rats. **(A)** Kidney tissue lysates were subjected to immunoblot analysis with specific antibodies against E-cadherin, vimentin or GAPDH. **(B)** Expression levels of E-cadherin **(B)** or vimentin **(C)** were quantified by densitometry and normalized to GAPDH. **(D)** Photomicrographs (original magnification, ×400) illustrate immunohistochemical staining of E-cadherin and vimentin in in kidney tissues. Graphic presentation of quantitative data for the staining of E-cadherin **(E)** and vimentin **(F)**. Data are represented as the mean ± SEM. *p < 0.05; **p < 0.01.

It has been reported that partial EMT occurs in renal tubular cells and contributes to renal interstitial fibrosis in CKD, including HN (Ryu et al., 2013; Stone et al., 2016; Wang et al., 2017). Partial EMT is characterized by loss of epithelial features with decreased expression of E-cadherin and acquired mesenchymal phenotype with increased expression of vimentin. As shown in Figures 4A–C, immunoblot analysis of the renal tissue indeed revealed that HN injury resulted in downregulation of the epithelial cell marker, E-cadherin and upregulation of vimentin, a mesenchymal marker, indicating that partial EMT of renal tubular cells occurs in HN. Treatment with I-BET151 blocked this response. Consistent with this observation, immunohistochemistry staining also showed that I-BET151 administration preserved E-cadherin expression levels and reduced expression of vimentin to the basal level in the kidney of HN (Figures 4D–F). Therefore, we suggest that I-BET151 inhibits the renal EMT that occurs in the kidney of HN rats.

### I-BET151 Abrogates the TGF-β/Smad3 Signaling Pathway in the Kidneys of HN Rats

**FIGURE 5:**
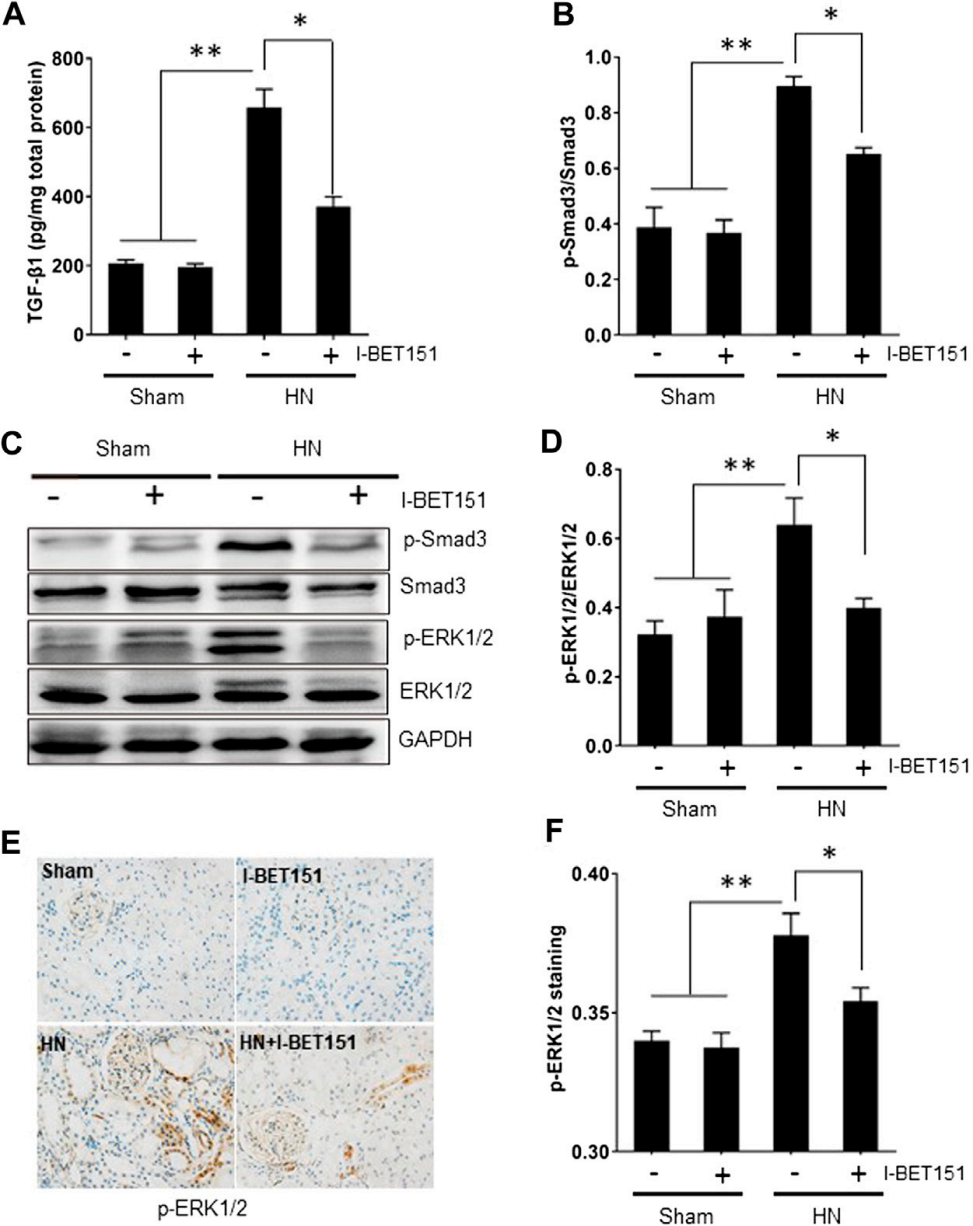
I-BET151 abrogates the TGF-β/Smad3 signaling pathway in kidneys of HN rats. **(A)** Protein was extracted from kidneys and subjected to measurement of TGF-β1 by ELISA as indicated. **(B)** Expression levels of p-Smad3 were quantified by densitometry and normalized to Smad3. **(C)** The kidney tissue lysates were subjected to immunoblot analysis with specific antibodies against p-Smad3, Smad3, p- ERK1/2, ERK1/2, or GAPDH. **(D)** Expression levels of P-ERK1/2 were quantified by densitometry and normalized to ERK1/2. **(E)** Photomicrographs (original magnification, ×400) illustrate immunohistochemical staining for p-ERK1/2 in kidney tissues. **(F)** p-ERK1/2 staining graphic presentation of quantitative data. Data are represented as the mean ± SEM. *p < 0.05; **p < 0.01.

TGF-β1 is the most potent fibrogenic factor and induces tissue fibrosis via activation of Smad3 (Lan, 2011; Ding et al., 2014; Meng et al., 2015). ERK1/2 also contributes to hyperuricemia-mediated renal injury (Liu et al., 2017b). To explore the effect of I-BET151 on the activation of TGF-β/Smad3 and ERK1/2 signaling pathways in rats with HN, we first examined the effect of I-BET151 on TGF-β1 expression in the kidney of HN rats by ELISA. Figure 5A shows that TGF-β1 expression was increased in the kidney of HN rats, and I-BET151 treatment decreased its expression. Next, we examined the effect of I-BET151 on phosphorylation of Smad3 and ERK1/2 in the kidney of HN rats. Western blot analysis of kidney lysates indicated that hyperuricemic-induced injury resulted in phosphorylation of Smad3 and ERK1/2, which was inhibited by I-BET151 treatment, whereas expression of total Smad3 and ER1/2 remained the same in all groups (Figures 5B–D). In addition, immunochemical staining showed that HN-induced p-ERK1/2 was mainly located in the glomerulus and renal tubules and I-BET151 reduced its expression (Figures 5E,F). Based on these findings, our results suggest that I-BET151 can inhibit activation of TGF-β/Smad3 and ERK1/2 signaling pathway in the kidney of HN rats.

### I-BET151 Suppressed the Activation of NF-κB, Expression of Proinflammatory Cytokines/Chemokines and Infiltration of Mononuclear Cells in the Kidney of HN Rats

**FIGURE 6:**
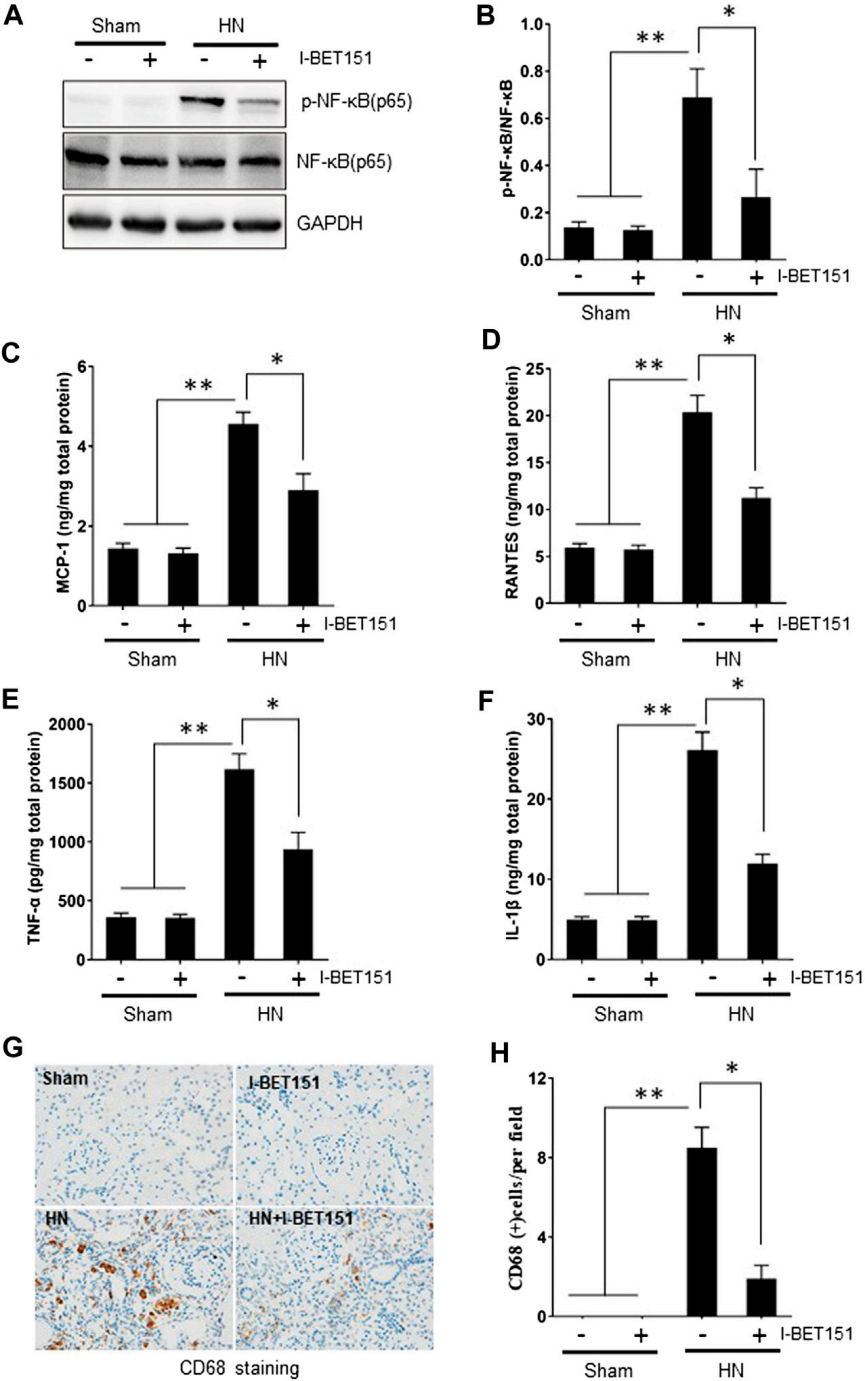
I-I-BET151 suppresses the activation of NF-κB and expression of proinflammatory cytokines/chemokines and infiltration of mononuclear cells in the kidney of HN rats **(A)** Kidney tissue lysates were subjected to immunoblot analysis with specific antibodies against p-NF-κB (p65), NF-κB (p65), or GAPDH. **(B)** Expression levels of p-NF-κB (p65) were quantified by densitometry and normalized to NF-κB (p65). Protein levels of **(C)** MCP-1, **(D)** RANTES, **(E)** TNF-α, and **(F)** IL-1β were measured by the ELISA. **(G)** Photomicrographs (original magnification, ×400) illustrate immunohistochemical staining of CD68 in kidney tissues. **(H)** CD68 staining graphic presentation of quantitative data. Data are represented as the mean ± SEM. *p < 0.05 vs. sham group and sham + I-BET151 group. **p < 0.05 vs. HN group.

Hyperuricemia-induced inflammation is associated with kidney injury (Jalal et al., 2013; Spiga et al., 2017). NF-κB is a key transcription factor that can drive expression of numerous proinflammatory cytokines/chemokines (Huang et al., 2013; Xu et al., 2016). To investigate whether I-BET151 would suppress the activation of NF-κB and inflammatory response in the kidney of HN rats, we examined the expression of phosphorylated NF-κB (p-NF-κB) by immunoblot blot, expression of some proinflammatory cytokines/chemokines by ELISA assay and mononuclear cell infiltration by staining CD68, a marker of activated macrophages. Figure 6 showed that HN injury induced phosphorylation of NF-κB, upregulation of MCP-1, RANTES, ICAM-1, TNF-α, and IL-1β as well as increased CD68-positive macrophages in the kidney of HN rats. I-BET151 treatment remarkedly suppressed NF-κB phosphorylation without altering expression of total NF-κB, reduced expression levels of all above mentioned cytokines/chemokines, and mononuclear cell infiltration. Collectively, our data suggest that I-BET151 can effectively inhibit HN-induced inflammatory responses in rats.

### I-BET151 Treatment Affects Neither Serum Xanthine Oxidase Activity nor Urate Transporters in HN Rats

**FIGURE 7:**
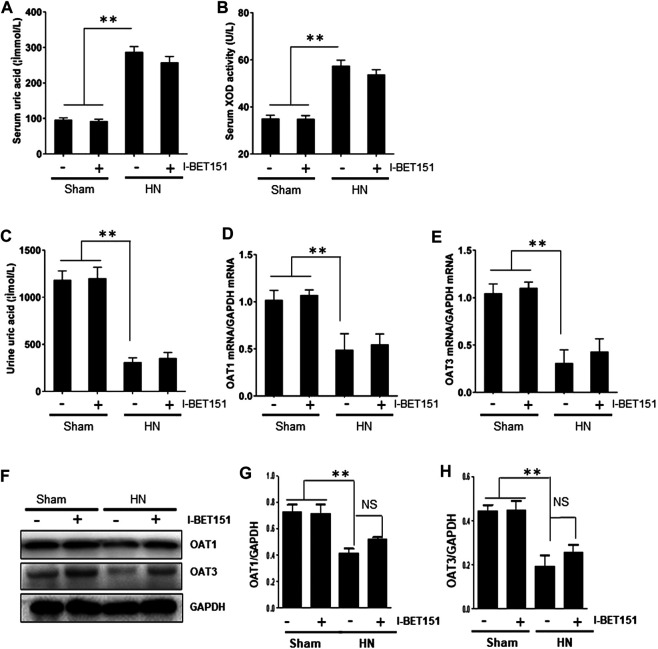
I-BET151 reduces serum uric acid and XOD activity and promotes uric acid excretion in HN rats. Levels of serum uric acid **(A)** and urine uric acid **(C)** were examined using an automatic biochemical assay. **(B)** Serum XOD activity was examined using a XOD kit. **(D)** OAT1 and **(E)** OAT3 mRNA expression in renal tissue from the different groups were determined by RT-PCR and normalized to GAPDH). Kidney tissue lysates were subjected to immunoblot analysis with specific antibodies against OAT1, OAT3 and GAPDH **(F)**. Expression levels of OAT1 **(G)** and OAT3 **(H)** were quantified by densitometry and normalized to GAPDH. Data are represented as the mean ± SEM. **p < 0.01.

Hyperuricemia is linked with upregulation of serum XOD activity, an enzyme that catalyzes the oxidation of hypoxanthine to xanthine and can further catalyze the oxidation of xanthine to uric acid, and downregulation of organic anion transporter (OAT) 1 and 3 that are involved in renal excretion of uric acid (Wu et al., 2017). As such, we further examined the effect of I-BET151 on the serum and urine uric acid levels and serum XOD activity and renal expression of OAT in HN rats. As shown in Figures 7A–H, serum levels of uric acid and XOD activity were increased whereas urine levels of uric acid as well as renal gene and protein levels of OAT 1 and OAT3 were reduced in HN rats; I-BET151 administration did not significantly affect changes of them. Therefore, it seems that neither production nor excretion of uric acid is subject to the regulation of I-BET151 targeted BET proteins.

## Discussion

The family of BET proteins functions as epigenetic readers and regulate expression of many genes associated with renal and other diseases (Ferri et al., 2016; Morgado-Pascual et al., 2019). In the current study, we evaluated the effects of BET protein inhibition by using I-BET151, a small-molecule BET inhibitor, on the development of HN and the mechanisms involved. Our results show that I-BET151 treatment improved renal function, reduced urine microalbumin levels, and alleviated interstitial fibrosis and inflammatory responses in the kidney of HN rats. BET inhibition also inhibited hyperuricemia–induced activation of TGF-β/Smad3, NF-κB and ERK1/2 signaling pathways. To our knowledge, this study is the first to demonstrate the effects of BET inhibition on the development and progression of HN.

BET proteins are frequently deregulated in disease states, in particular cancer, and contribute to aberrant chromatin remodeling and gene transcription (Morgado-Pascual et al., 2019) (Ferri et al., 2016). Although BRD2, BRD3, and BRD4 have been documented to be ubiquitously expressed (Morgado-Pascual et al., 2019), our data demonstrated that Brd3 and Brd4, but not Brd2 were expressed in the normal kidney of rats by immunoblot analysis. In response to hyperuricemia, renal expression of Brd2 was induced, Brd4 upregulated, but Brd3 expression was not altered; administration of I-BET151 abolished expression of Brd2 and Brd4, but did not affect Brd3 expression in the kidney of HN. Coincident with Brd2 and Brd4 repression, I-BET151 improved renal function and attenuated renal fibrosis. Therefore, it appears that Brd2 and Brd4 are BET proteins critically involved in the pathogenesis of HN. In support to this conclusion, we have recently demonstrated that transfection of siRNA specific to Brd4 inhibits serum- or TGF-β1-induced activation of renal interstitial fibroblasts in culture (Xiong et al., 2016). Additional investigations are needed to elucidate the role of individual BET proteins in HN and other kidney diseases by genetic approaches. Currently, it remains unclear about the mechanism of I-BET151 elicited decline of Brd2 and Brd4 expression. Since BET proteins can recognize acetylated lysine residues and interact with many chromatin remodeling proteins and some transcriptional factors, it is anticipated that the interaction of Brd2 and Brd4 with transcriptional factors and histone modifiers would initiate a signal that regulates expression of these two BET proteins through a feed-back loop, while inhibition of such interactions may block this process, leading to downregulation of Brd2 and Brd4.

The mechanism by which I-BET151 attenuates pathogenesis of HN may be multifaceted. It is well known that transition of renal epithelial cell type to a mesenchymal type is an essential step for the initiation and progression of renal fibrosis, and uric acid can induce phenotypic transition of renal tubular cells via decreased synthesis and enhanced degradation of E-cadherin (Ryu et al., 2013). In this study, we found that I-BET151 treatment prevented downregulation of E-cadherin and inhibited upregulation of vimentin, a mesenchymal marker, in the kidneys of HN rats. In line with this observation, I-BET151 also inhibited activation of renal interstitial fibroblasts as evidenced by decreased expression levels of α-SMA, and diminished expression of ECM proteins, including collagen I and fibronectin, in the kidney of HN rats. As such, we suggest that I-BET151 may inhibit hyperuricemia-induced transition of renal epithelial cell to a profibrotic phenotype, and subsequently decreased production and release of some profibrotic growth factors/cytokines that stimulate fibroblast activation and excessive production of ECM proteins. In addition, although administration of I-BET151 reduced hyperuricemia-induced pathological changes, it did not significantly affect serum uric acid levels in HN rats. This suggests that I-BET151 may protect against HN through attenuating uric acid-induced renal injury via interfering with some key profibrotic signaling pathways rather than reducing serum uric acid levels.

A key signaling pathway that mediates profibrotic response of renal epithelial cells and activation of renal fibroblast is the TGFβ/Smad3 (Meng et al., 2015; Gu et al., 2020). In this study, we demonstrated that treatment with I-BET151 diminished hyperuricemia-induced TGF-β1 expression and phosphorylation of Smad3. In agreement with this observation, I-BET151 also effectively suppressed activation of Smad3 signaling in a murine model of UUO-induced renal fibrosis (Xiong et al., 2016). These data strongly suggest that I-BET-151 elicited inhibition of HN is associated with inactivation of the TGFβ/Smad3 signaling pathway. Given that TGF-β1 is the primary ligand that interacts with TGF receptors and then initiates activation of Samd3, it is possible that I-BET151 targeted BETs is coupled to the regulation of TGF-β1 expression and production. In this regard, we observed that I-BET151 was effective in suppressing phosphorylation of ERK1/2, a critical intracellular signaling molecule that drives TGF-β1 expression (Morita et al., 2019). In addition, as ERK1/2 can interact with Smad3 to TGF-β1-stimulated multiple biological functions including renal fibrosis (Nakagawa et al., 2005), I-BET151 may also inhibit renal fibrosis through interfering with ERK1/2-mediated regulation of Smad3 and other profibrotic machinery downstream of TGFβ receptors. The detailed mechanism by which BET proteins regulate the activation of TGFβ/Smad3 signaling needs further investigations.

The BET protein has been shown as an important coactivator of the inflammatory transcriptional program (Zou et al., 2014). NF-κB-mediated signaling promotes the transcription of pro-inflammatory cytokines/chemokine such as IL-1, IL-6, TNF-α and MCP1 and plays a crucial role in the inflammatory response induced by uric acid (Zhou et al., 2012; Liu et al., 2017a). It has been reported that Brd4 binding to acetylated lysine-310 of RelA is essential to activate specific NF-κB target genes (Huang et al., 2009; Zou et al., 2014); pharmacological inhibition of BET could thus be a unique pharmacological strategy to block NF-κB activation via disrupting the interaction of Brd4 with nuclear acetylated-RelA. In this study, we indeed demonstrated that I-BET151 treatment inhibited phosphorylation of NF-κBp65 and expression of MCP-1, RANTES, TNF-α, and IL-1β in the kidney of HN rats. In addition, I-BET151 was also effective in reducing macrophage infiltration into the injured kidney. This suggests that suppression of proinflammatory responses would be another important mechanism by which I-BET151 attenutes development and progression of HN.

Hyperuricemia is an independent risk factor of renal damage and can exacerbate the progression of CKD (Srivastava et al., 2018). Previous studies have demonstrated that uric acid–lowering therapy may retard the progression of CKD (Bose et al., 2014; Sampson et al., 2017). Serum uric acid levels are largely determined by uric acid production and renal excretion in urine. As such, we evaluated the effects of I-BET151 on the activity of serum XOD, a fundamental enzyme promoting uric acid production and expression of OAT1 and OAT3, two organic anion transporters associated with renal excretion of uric acid. Our results show that the activity of serum XOD was increased whereas renal OAT1 and OAT3 were reduced in HN rats, however, I-BET151 treatment did not significantly reduce the activity of serum XOD in HN rats and increased expression of OAT1 and OAT3. These results suggest that I-BET151-eiliciated renoprotection is not associated with alteration of serum uric acid levels.

Numerous studies have implicated the BET bromodomain family of proteins in regulating biological processes such as inflammation and tissue fibrosis. Given that BET proteins can bind to acetylated residues in histone and some transcription factors, leading to their activation, therapies aimed at inhibiting BET protein function may represent a novel therapeutic strategy for chronic kidney disorders including HN. Recently, several small molecule inhibitors of BET proteins have been developed and demonstrate anti-fibrotic properties in liver (Ding et al., 2015), lung (Sanders et al., 2020) (Wang et al., 2018), kidney (Xiong et al., 2016) (Zhou et al., 2017) and heart (Duan & McMahon, 2017). Human phase I clinical trials also demonstrate that BET inhibitors have minimal and reversible clinical toxicity in human cancer patients (Boi et al., 2015). Most adverse effects are nausea, fatigue and other significant observed toxicities include asthenia, anorexia and reversible thrombocytopaenia (Morgado-Pascual et al., 2019; Ameratunga & Braña, 2020). Currently, more than twenty clinical trials are underway to evaluate the efficacy of BET inhibitors in tumors; two trials are testing this class of drugs for cardiovascular diseases and CKD (NCT02586155 and NCT03160430). As such, BET inhibitors may be developed as treatments for HN and other chronic fibrotic kidney diseases.

## Conclusion

Our results demonstrate that I-BET151 alleviates the progression of HN in a rat model. This effect was associated with inhibition of TGF-β signaling, renal tubular EMT and inflaammation, but not related to reduction of serum uric acid levels. Thus, BET inhibition may have therapeutic potential for the prevention and treatment of HN.

## Data Availability

The raw data supporting the conclusions of this article will be made available by the authors, without undue reservation.
